# Supplementary material for: Empirical methods for controlling false positives and estimating confidence in ChIP-Seq peaks
Source: BMC Bioinformatics. 2008 Dec 5;9:523. doi: 10.1186/1471-2105-9-523 (PMC2628906; doi:10.1186/1471-2105-9-523)
Supplement: Additional File 1 — A variety of html documents from the USeq web site detailing the available applications, their best usage, output file type descriptions, command line menus, etc. [file 1471-2105-9-523-S1.zip › USeqUserGuides/JavaDoc.zip!/JavaDoc/trans/main/PeakPicker.html]

PeakPicker


|  |  |  |
| --- | --- | --- |
| **Overview** \| **Package** \| **Class** \| **Use** \| **Tree** \| **Deprecated** \| **Index** \| **Help** | |  |
| **PREV CLASS**   **NEXT CLASS** | **FRAMES**    **NO FRAMES**     **All Classes** |  |
| SUMMARY: NESTED \| FIELD \| CONSTR \| METHOD | DETAIL: FIELD \| CONSTR \| METHOD |  |


---


## trans.main Class PeakPicker

```
java.lang.Object
  trans.main.PeakPicker
```

---

``` public class PeakPicker extends java.lang.Object ```

Finds peaks in `Interval`s, both flat topped or peaks with sloping sides.

---

**Constructor Summary**

`PeakPicker()`


| **Method Summary** | |
| --- | --- |
| `double` | `averageWindowScores(int start, int stop)`             Averages the smoothed oligo scores, stop is inclusive. |
| `boolean` | `checkWidth(int leftIndex, int peakIndex, int rightIndex)` |
| `boolean` | `expandFlatTopPeak()`             Attempts to expand the leftIndex and rightIndex |
| `int` | `findFlatTopPeak()` |
| `int` | `findHighestScore()` |
| `int` | `findPeak()` |
| `int` | `findPeakBp(int peakIndex)` |
| `Interval` | `getInterval()` |
| `int` | `getLeftIndex()` |
| `int` | `getMaxNumPeaks()` |
| `int` | `getMinBpSides()` |
| `double` | `getMinPercent()` |
| `int` | `getMinRun()` |
| `int` | `getNumOligos()` |
| `Oligo[]` | `getOligos()` |
| `int` | `getPeakBp()` |
| `int` | `getPeakIndex()` |
| `int` | `getRightIndex()` |
| `double` | `getScoreCutOff()` |
| `int` | `growLeft(int index)`             Uses the idea of a local window, counts the number of pos slopes within the window, if > minPercent, keeps advancing. |
| `int` | `growRight(int index)`             Uses the idea of a local window, counts the number of neg slopes within the window, if > minPercent, keeps advancing. |
| `void` | `mask(int start, int stop)` |
| `void` | `pickPeaks(Interval[] intervals)` |
| `void` | `setInterval(Interval interval)` |
| `void` | `setLeftIndex(int leftIndex)` |
| `void` | `setMakeFlattops(boolean makeFlattops)` |
| `void` | `setMaxBpGap(int maxBpGap)` |
| `void` | `setMaxNumPeaks(int maxNumPeaks)` |
| `void` | `setMaxScoreFractionDrop(double maxScoreFractionDrop)` |
| `void` | `setMinBpSides(int minBpSides)` |
| `void` | `setMinPercent(double minPercent)` |
| `void` | `setMinRun(int minRun)` |
| `void` | `setNumOligos(int numOligos)` |
| `void` | `setOligos(Oligo[] oligos)` |
| `void` | `setPeakBp(int peakBp)` |
| `void` | `setPeakIndex(int peakIndex)` |
| `void` | `setRightIndex(int rightIndex)` |
| `void` | `setScoreCutOff(double scoreCutOff)` |
| `boolean` | `setSubWindowScoreAsMinScore()`             Averages the smoothed scores in the best sub window. |
| `void` | `slopeOligos()` |

**Methods inherited from class java.lang.Object**

`equals, getClass, hashCode, notify, notifyAll, toString, wait, wait, wait`

**Constructor Detail**

### PeakPicker

```
public PeakPicker()
```


**Method Detail**

### expandFlatTopPeak

```
public boolean expandFlatTopPeak()
```

:   Attempts to expand the leftIndex and rightIndex

---


### averageWindowScores

```
public double averageWindowScores(int start,
                                  int stop)
```

:   Averages the smoothed oligo scores, stop is inclusive.

---


### setSubWindowScoreAsMinScore

```
public boolean setSubWindowScoreAsMinScore()
```

:   Averages the smoothed scores in the best sub window. and sets this \* maxScoreFractionDrop as the scoreCutOff.

---


### pickPeaks

```
public void pickPeaks(Interval[] intervals)
```

---


### mask

```
public void mask(int start,
                 int stop)
```

---


### findFlatTopPeak

```
public int findFlatTopPeak()
```

---


### findPeak

```
public int findPeak()
```

---


### checkWidth

```
public boolean checkWidth(int leftIndex,
                          int peakIndex,
                          int rightIndex)
```

---


### findPeakBp

```
public int findPeakBp(int peakIndex)
```

---


### growRight

```
public int growRight(int index)
```

:   Uses the idea of a local window, counts the number of neg slopes within the window, if > minPercent, keeps advancing.

---


### growLeft

```
public int growLeft(int index)
```

:   Uses the idea of a local window, counts the number of pos slopes within the window, if > minPercent, keeps advancing.

---


### findHighestScore

```
public int findHighestScore()
```

---


### slopeOligos

```
public void slopeOligos()
```

---


### getInterval

```
public Interval getInterval()
```

---


### setInterval

```
public void setInterval(Interval interval)
```

---


### getLeftIndex

```
public int getLeftIndex()
```

---


### setLeftIndex

```
public void setLeftIndex(int leftIndex)
```

---


### getMinBpSides

```
public int getMinBpSides()
```

---


### setMinBpSides

```
public void setMinBpSides(int minBpSides)
```

---


### getMinPercent

```
public double getMinPercent()
```

---


### setMinPercent

```
public void setMinPercent(double minPercent)
```

---


### getMinRun

```
public int getMinRun()
```

---


### setMinRun

```
public void setMinRun(int minRun)
```

---


### getNumOligos

```
public int getNumOligos()
```

---


### setNumOligos

```
public void setNumOligos(int numOligos)
```

---


### getOligos

```
public Oligo[] getOligos()
```

---


### setOligos

```
public void setOligos(Oligo[] oligos)
```

---


### getPeakBp

```
public int getPeakBp()
```

---


### setPeakBp

```
public void setPeakBp(int peakBp)
```

---


### getPeakIndex

```
public int getPeakIndex()
```

---


### setPeakIndex

```
public void setPeakIndex(int peakIndex)
```

---


### getRightIndex

```
public int getRightIndex()
```

---


### setRightIndex

```
public void setRightIndex(int rightIndex)
```

---


### getScoreCutOff

```
public double getScoreCutOff()
```

---


### setScoreCutOff

```
public void setScoreCutOff(double scoreCutOff)
```

---


### getMaxNumPeaks

```
public int getMaxNumPeaks()
```

---


### setMaxNumPeaks

```
public void setMaxNumPeaks(int maxNumPeaks)
```

---


### setMakeFlattops

```
public void setMakeFlattops(boolean makeFlattops)
```

---


### setMaxBpGap

```
public void setMaxBpGap(int maxBpGap)
```

---


### setMaxScoreFractionDrop

```
public void setMaxScoreFractionDrop(double maxScoreFractionDrop)
```


---


|  |  |  |
| --- | --- | --- |
| **Overview** \| **Package** \| **Class** \| **Use** \| **Tree** \| **Deprecated** \| **Index** \| **Help** | |  |
| **PREV CLASS**   **NEXT CLASS** | **FRAMES**    **NO FRAMES**     **All Classes** |  |
| SUMMARY: NESTED \| FIELD \| CONSTR \| METHOD | DETAIL: FIELD \| CONSTR \| METHOD |  |


---
